# Prognostic risk analysis related to radioresistance genes in colorectal cancer

**DOI:** 10.3389/fonc.2022.1100481

**Published:** 2023-01-18

**Authors:** Haoren Qin, Heng Zhang, Haipeng Li, Qiong Xu, Wanjun Sun, Shiwu Zhang, Xipeng Zhang, Siwei Zhu, Hui Wang

**Affiliations:** ^1^ Department of Oncology, Tianjin Union Medical Center, Nankai University, Tianjin, China; ^2^ School of Medicine, Nankai University, Tianjin, China; ^3^ School of Integrative Medicine, Tianjin University of Traditional Chinese Medicine, Tianjin, China; ^4^ Department of Pathology, Tianjin Union Medical Center, Nankai University, Tianjin, China; ^5^ Department of Colorectal Surgery, Tianjin Union Medical Center, Nankai University, Tianjin, China

**Keywords:** colorectal cancer, radiotherapy, radioresistance, prognostic analysis, tumor microenvironment

## Abstract

**Background:**

Radiotherapy (RT) is one of the most important treatments for patients with colorectal cancer (CRC). Radioresistance is the crucial cause of poor therapeutic outcomes in colorectal cancer. However, the underlying mechanism of radioresistance in colorectal cancer is still poorly defined. Herein we established a radioresistant colorectal cancer cell line and performed transcriptomics analyses to search for the underlying genes that contribute to radioresistance and investigate its association with the prognosis of CRC patients.

**Methods:**

The radioresistant cell line was developed from the parental HCT116 cell by a stepwise increased dose of irradiation. Differential gene analysis was performed using cellular transcriptome data to identify genes associated with radioresistance, from which extracellular matrix (ECM) and cell adhesion-related genes were screened. Survival data from a CRC cohort in the TCGA database were used for further model gene screening and validation. The correlation between the risk score model and tumor microenvironment, clinical phenotype, drug treatment sensitivity, and tumor mutation status were also investigated.

**Results:**

A total of 493 different expression genes were identified from the radioresistant and wild-type cell line, of which 94 genes were associated with ECM and cell adhesion-related genes. The five model genes *TNFRSF13C*, *CD36*, *ANGPTL4*, *LAMB3*, and *SERPINA1* were identified for CRC radioresistance *via* screening using the best model. A ROC curve indicated that the AUC of the resulting prognostic model (based on the 5-gene risk score and other clinical parameters, including age, sex, and tumor stages) was 0.79, 0.77, and 0.78 at 1, 2, and 3 years, respectively. The calibration curve showed high agreement between the risk score prediction and actual survival probability. The immune microenvironment, drug treatment sensitivity, and tumor mutation status significantly differed between the high- and low-risk groups.

**Conclusions:**

The risk score model built with five radioresistance genes in this study, including *TNFRSF13C*, *CD36*, *ANGPTL4*, *LAMB3*, and *SERPINA1*, showed favorable performance in prognosis prediction after radiotherapy for CRC.

## 1 Introduction

Colorectal cancer (CRC) a common malignant tumor of the digestive tract and the second leading cause of cancer death worldwide, which is a serious threat to human health (1). Radiotherapy is an important treatment for CRC and has become a standard treatment regimen for stage II and III CRC. According to the results of existing clinical trials, compared with preoperative radiotherapy or surgical treatment alone, postoperative radiotherapy has obvious advantages in tumor downstaging, pathological response rate, and progression-free survival. NCCN treatment guidelines have proposed preoperative radiotherapy combined with surgery as the recommended treatment mode for locally advanced CRC. However, preoperative radiotherapy for CRC patients still has the problem of low sensitivity. A clinical study showed that the pathologic complete response rate of preoperative radiotherapy in CRC was only 8%. Therefore, improving the radiotherapy sensitivity of CRC and identifying the mechanisms associated with radioresistance remain challenges in CRC treatment.

The direct and indirect destruction of DNA double strands by high-energy particle beams from irradiation and excess ROS generation are the main mechanisms by which radiotherapy kills tumor cells. However, at a limited radiation dose, tumor cells evolved to develop multiple mechanisms, including DNA damage response, cell cycle re-distribution, and ROS detoxification, to avoid the cytotoxicity of ionizing radiation (2–4). The surviving tumor cells in this process may develop a certain radioresistance, with great impact on the prognosis of patients with CRC (5). Notably, in addition to tumor cells themselves, the microenvironment of these cells, i.e., the tumor microenvironment, also plays a crucial role in the efficacy of radiotherapy. The tumor microenvironment is composed of multiple components, including immune cells, fibroblasts, and extracellular matrix. Studies showed that in the presence of abundant cytotoxic T-lymphocyte (CTL) infiltration in the tumor microenvironment, the radiation dose necessary to control tumor progression was significantly reduced. Conversely, in the absence of CTL infiltration in the tumor microenvironment, larger doses are required to inhibit tumor growth, suggesting that the activation of the immune system enhances malignancy responsiveness to radiotherapy.

However, despite the fact that radiotherapy could induce immunogenic cell death to initiate anti-tumor immune response, changes in other components in the tumor microenvironment and their effects on tumor growth are still scarcely researched. The extracellular matrix (ECM) is an integral part of the tumor microenvironment, which is composed of multiple components, including collagen, elastin, proteoglycan, and glycoprotein. Abnormal expression of ECM components and remodeling of ECM during tumor progression can promote drug tolerance and transformation and metastasis of tumor cells. On the one hand, cells can integrate signals from ECM to modify their functionalities and behaviors. On the other hand, cells within the tumor environment also remodel ECM by synthesizing and secreting matrix macromolecules under the control of multiple extracellular signals, which leads to the reformation of the biophysical and biochemical properties of ECM (6). Radiotherapy can remodel ECM by inducing loss of hyaluronic acid and collagen synthesis. In turn, remodeled ECM can also improve the conditions of cell growth, cell differentiation, and survival of tissues, which further leads to radioresistance in tumor cells. However, the mechanism underlying the reciprocal communication between tumor cells and ECM is complex, and the hallmarks leading to radioresistance and can predict CRC radiosensitivity and prognosis remain poorly understood.

To further expand the study of the molecular mechanisms of radioresistance and its impact on patient prognosis, we developed radiation-tolerant CRC cell lines. Differential gene expression analysis was performed on the RNA-seq data of tolerant and wild-type controls to identify the genes associated with radioresistance; these genes were then screened for association with ECM and with cell adhesion, and analyzed using survival data from the TCGA-COAD (Colon Adenocarcinoma) cohort in the TCGA database. Our results show that the combined risk scores of the five marker genes identified from the screening procedures can achieve a more accurate prediction of prognosis after CRC radiotherapy, thereby acting as molecular indicators in the field of CRC radiotherapy.

## 2 Materials and methods

### 2.1 Study design and data source

Differential gene expression analysis was performed using sequencing data from the human colon cancer cells HCT-116 (purchased from the Shanghai Institutes for Biological Sciences, Chinese Academy of Sciences, Shanghai, China), which consisted of three wild-type (HCT116^WT^) and three radioresistant (HCT116^RR^) cell lines, to identify genes associated with radioresistance. ECM and cell adhesion-related genes were screened, and the survival data from the COAD colon cancer cohort in the TCGA database were used for analysis (Scheme 1).

The TCGA-COAD cohort data were downloaded from the UCSC Xena website (http://xena.ucsc.edu/). The data used in the analysis included gene expression data from 471 tumor tissues, survival data from 454 patients, clinical phenotype data from 478 patients, and tumor mutation data from 399 patients. Risk scores were validated using an external dataset, GSE40967 (7), which was designed to validate whether analytically constructed risk scores could predict prognostic risk in a separate cohort of patients. This dataset contains survival and gene expression assay data for 562 patients.

### 2.2 Establishment of CRC radioresistant cell line

HCT116 cells were exposed to a linear accelerator (Varian Clinic 21 ES; Varian Medical Systems, Crawley, UK), which generates high-energy X-rays (6 MeV) at 0.99 Gy/min. Radioresistant cell lines (HCT116 RR) were developed from the parental cell line (HCT116) by increasing the X-ray dose of fraction irradiation stepwise from 0.5 to 2 Gy/day *in vitro* (2). Cells were initially exposed to 0.5 Gy/day of X-rays for 5 days. Subsequently, the cells were exposed to 1 Gy/day of X-rays for 10 days. Thereafter, the surviving cells were exposed to 1.5 Gy/day of X-rays for 15 days. Cells that could proliferate under exposure to 1.5 Gy/day of X-rays were further exposed to 2 Gy/day of X-rays. If these cells proliferated constantly under exposure to 2 Gy/day of X-rays for more than 30 days, it was determined that a HCT116 RR had been obtained.

### 2.3 RNA extraction, library preparation, and sequencing

Total RNA was extracted from the HCT116 cell line using TRIzol^®^ Reagent according the manufacturer’s instructions (Magen). RNA samples were detected based on the A260/A280 absorbance ratio with a Nanodrop ND-2000 system (Thermo Scientific, USA), and the RIN of RNA was determined by an Agilent Bioanalyzer 4150 system (Agilent Technologies, CA, USA). Only qualified samples were used for library construction.

Paired-end libraries were prepared using a ABclonal mRNA-seq Lib Prep Kit (ABclonal, China) following the manufacturer’s instructions. The mRNA was purified from 1 μg of total RNA using oligo (dT) magnetic beads followed by fragmentation carried out using divalent cations at elevated temperatures in ABclonal First Strand Synthesis Reaction Buffer. Subsequently, first-strand cDNAs were synthesized with random hexamer primers and Reverse Transcriptase (RNase H) using mRNA fragments as templates, followed by second-strand cDNA synthesis using DNA polymerase I, RNAseH, buffer, and dNTPs. The synthesized double stranded cDNA fragments were then adapterligated for preparation of the paired-end library. Adaptor-ligated cDNA was used for PCR amplification. PCR products were purified (AMPure XP system), and library quality was assessed on an Agilent Bioanalyzer 4150 system. Finally, the library preparations were sequenced on an Illumina Novaseq 6000 (or MGISEQ-T7), and 150-bp paired-end reads were generated.

### 2.4 Identification of differentially expressed genes

Raw data in fastq format were firstly processed through in-house perl scripts. In this step, the adapter sequence was removed and low quality reads were filtered out (the number of lines with a string quality value less than or equal to 25 accounted for more than 60% of the entire reading and the N ratio (i.e., base information could not be determined) was greater than 5% of total reads) to obtain clean reads that could be used for subsequent analysis. Then, the clean reads were separately aligned to the reference genome in the orientation mode using the HISAT2 software (http://daehwankimlab.github.io/hisat2/) to obtain mapped reads. FeatureCounts (http://subread.sourceforge.net/) was used to count the read numbers mapped to each gene. Then, the FPKM of each gene was calculated based on the length of a gene and the read count mapped to that gene. Differentially expressed genes (DEGs) were identified using the “DESeq2” R package. Adjusted p-value < 0.05 and |log_2_FC| > 1 were used as cut-off points to identify DEGs for subsequent analysis.

### 2.5 Identification of DEGs associated with survival and establishment of a prognostic gene signature

The candidate genes were used to generate prognosis-related risk scores. The patients in the TCGA-COAD cohort with both gene expression information from tumor samples and complete survival information served as the training set. Univariate Cox regression analysis was used to screen for DEGs in the training set that were strongly associated with patient survival, using a p-value < 0.05. Next, LASSO regression was applied to further establish radioresistance-related risk profiles. The prognostic risk score model was established with the following formula: risk score = expression level of Gene1 × β1 + expression level of Gene2 × β2 +…+ expression level of Genen × βn (where β is the regression coefficient calculated by the LASSO regression). Risk scores were calculated for each patient using a risk-score model. The samples were assigned to high-risk or low-risk groups according to the median risk score. Kaplan–Meier curves were used to compare the differences in overall survival (OS) between the high-risk and low-risk groups. ROC curves for the 1-, 3-, 5- and 7-year OS were generated for the two groups. The established risk score was evaluated in an external independent set, GSE40967, to assess its performance in prognosis prediction.

### 2.6 Bioinformatics analysis for differentially expressed genes

#### 2.6.1 Functional enrichment of differential genes

We performed gene ontology (GO) and Kyoto Encyclopedia of Genes and Genomes (KEGG) pathway analyses and visualized the results using the R package “clusterProfiler” (8) to determine the functional role of differentially expressed radioresistance-related genes. A p-value < 0.05 for GO terms or KEGG pathways was considered statistically significant.

#### 2.6.2 Protein-protein interaction network

A protein–protein interaction (PPI) network for differential genes was constructed using STRING v3.9.1 (http://string-db.org), a search tool for studying gene interactions (9). The minimum interaction score was set at greater than 0.4, and isolated nodes in the network were removed.

#### 2.6.3 Gene set enrichment analysis

GSEA is a method that uses genes from a pre-defined gene set to assess distribution trends in a gene list ranked with phenotypic relevance to judge their contribution to phenotype. The “GSEA” R package was used to find the function and pathway associated with the high- and low-risk groups.

#### 2.6.4 Immune microenvironment analysis

Immune and stromal scores predict the amount of immune and stromal components in a tumor or disease. The immune and stromal scores of COAD samples were calculated using the ESTIMATE algorithm available in the R package “ESTIMATE” (10). The ssGSEA (Single Sample Gene Set Enrichment Analysis, ssGSEA) algorithm (11) was used to assess the proportion of 28 immune cell subtypes in the high- and low-risk groups.

#### 2.6.5 Prediction of treatment sensitivity and analysis of tumor mutation status in patients with different risk scores

We compared the expression of 34 immune checkpoint genes and 23 HLA family genes between the high- and low-risk groups. The potential patient response to immunotherapy was inferred from the tumor immune dysfunction and rejection (TIDE). The “maftools” R package was applied to analyze and visualize somatic mutation data and to calculate the tumor mutation burden (TMB) score for individuals in the TCGA dataset.

### 2.7 Construction and evaluation of predictive nomograms

Independent prognostic factors selected by univariate and multivariate Cox regression analyses were used to construct nomograms to assess the probability of OS. Subsequently, calibration curves were used to estimate whether the predicted survival outcome was close to the actual outcome. DCA was performed to confirm the clinical utility of the nomogram model.

### 2.8 Statistical analysis

Analyses were performed in the R software (R software, version 4.1.0). The R and package versions can be found in **Supplementary Figure S2**. The Wilcoxon test was used for statistical comparisons between two groups, the Kruskal–Wallis test for statistical comparisons between multiple groups. The Bonferroni method was used to correct for multiple comparisons. An adj.p-value < 0.05 was regarded as statistically significant. Cox regression analysis was performed for outcomes adjusted with covariates for which the *p*-value was <0.05 in a univariable Cox analysis. the Spearman correlation coefficient for correlations between two continuous variables, except for group comparisons specific to high-throughput data.

## 3 Results

### 3.1 Identification of differential genes in the radioresistant cell line HCT116^RR^ compared to HCT116^WT^

**Figure 1:**
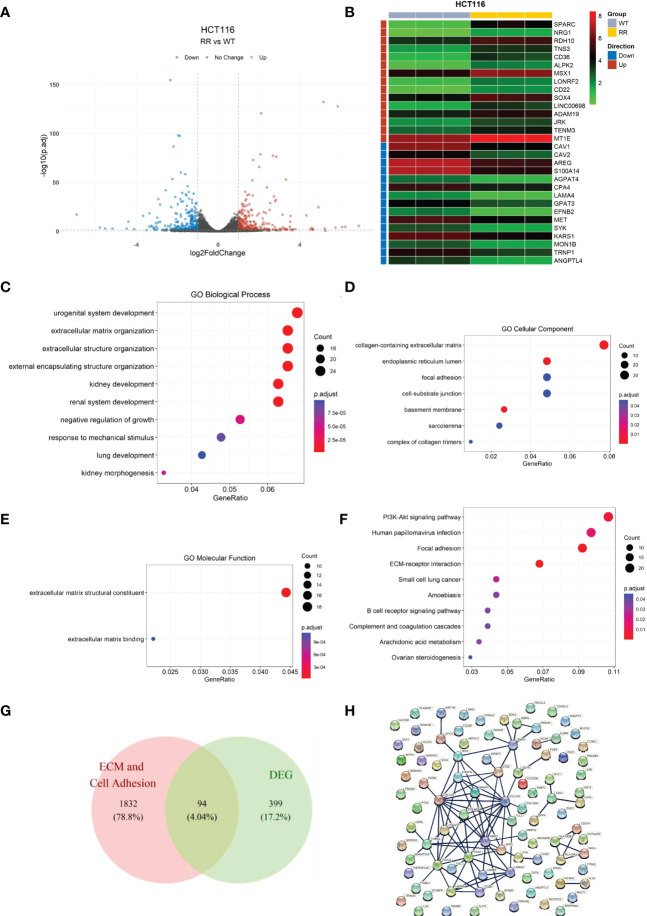
Identification of differentially expressed genes in radiotherapy-tolerant cell lines RR and WT compared to those in WT in HCT-116 cells. **(A)** Volcano plot of differential gene expression analysis. **(B)** Heat map of differentially expressed genes. **(C)** Differentially expressed gene enrichment results: GO biological processes. **(D)** Differentially expressed gene enrichment results: GO cellular components. **(E)** Differentially expressed gene enrichment results: GO molecular functions. **(F)** Differentially expressed gene KEGG enrichment results. **(G)** Candidate gene screening Wayne diagram. **(H)** Candidate gene protein interactions network.

We first verified the radioresistance of the HCT116^WT^ and the HCT116^RR^ cell line using clone formation assay (**Supplementary Figure S1**). We first screened for DEGs using the HCT-116 radiotherapy-tolerant cell line HCT116^RR^ and the HCT116^WT^ cell line as comparative samples. A total of 493 DEGs (RR vs. WT) were identified, of which 259 genes were upregulated and 234 genes were downregulated (**Supplementary Table S1**), using a p < 0.05 and |log_2_FC|>1 as thresholds (**Figure 1A**). The top-15 upregulated and downregulated genes were arranged according to the p-value and visualized using a heat map (**Figure 1B**).

GO enrichment analysis (**Figures 1C–E** and **Supplementary Table S2**) showed that the DEGs were mainly enriched in the biological processes “extracellular matrix organization”, “extracellular structure organization”, and “external encapsulating structure organization”. In cellular the components and molecular function categories, the DEGs were mainly enriched in the functions “collagen-containing extracellular matrix”, “focal adhesion”, “cell-substrate junction”, “extracellular matrix structural constituent”, and “extracellular matrix binding”, which indicates that the differential genes contribute the most to the functions of extracellular matrix structural constituents and extracellular matrix binding.

Similarly, KEGG enrichment results (**Figure 1F** and **Supplementary Table S3**) showed that the DEGs contained the two pathways of focal adhesion and ECM-receptor interaction. Combining the GO and KEGG enrichment results, we determined that the DEGs were mainly enriched for cell adhesion and ECM-related functions and pathways. Therefore, the cell adhesion and ECM-related DEGs were screened as candidate genes for subsequent analyses.

We cross-referenced the cell adhesion and extracellular matrix-related genes with the above-mentioned DEGs by releasing all genes containing the following annotations or their subdivisions: GO:0031012 extracellular matrix; GO:0030198 GO:0031012 extracellular matrix; GO:0030198 extracellular matrix organization; GO:1903053 regulation of extracellular matrix organization; GO:0035426 extracellular matrix-cell signaling; GO:0007155 cell adhesion; GO:0030155 regulation of cell adhesion; GO:0007160 cell-matrix adhesion; and GO:0050840 extracellular matrix binding. A total of 94 overlapping genes from DEGs and ECM-related genes were retained as candidate genes for subsequent analysis (**Figure 1G**). For these 94 genes, the interaction network of the encoded proteins was mapped using the STRING database (**Figure 1H**).

### 3.2 Establishment and validation of prognostic risk score

**Figure 2:**
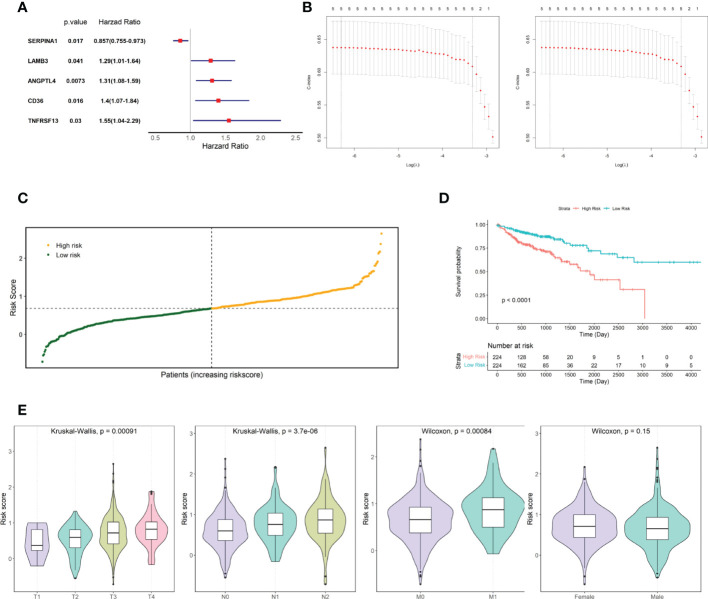
Screening for prognosis-associated genes. **(A)** Prognosis-associated gene Hazard Ratio display. **(B)** LASSO regression analysis. **(C)** TCGA COAD cohort risk score distribution. **(D)** KM curves for high- and low-risk groups in the TCGA COAD cohort. **(E)** Risk score distribution of patients at different TNM stages and different genders.

The relationship between candidate gene expression and the OS of patients was analyzed using a one-way Cox regression model. Five genes, *TNFRSF13C*, *CD36*, *ANGPTL4*, *LAMB3*, and *SERPINA1*, were found to be associated with OS, as shown in **Figure 2A**. LASSO regression analysis was performed using these five prognostic genes (**Figure 2B**), and the best model parameter was λ=0.001838643. The linear combination of the five genes and their coefficients in the model were used as the risk score, and the coefficients of the five genes are shown in **Table 1**.

**Table 1:** Gene coefficients of model genes.

Model genes	Gene coefficient
TNFRSF13C	0.238733819
CD36	0.174308895
ANGPTL4	0.218230164
LAMB3	0.21302476
SERPINA1	–0.210615904

Risk scores were calculated for patients in the TCGA COAD cohort, and patients were divided into two groups based on the median risk score: high and low risk (**Figure 2C**). The risk score distribution and associated survival status implied that there were more dead patients with an increase in the risk score. Furthermore, survival analysis showed that there was a significant difference between the low- and high-risk groups (p-value < 0.0001, **Figure 2D**). After statistically testing whether there were differences in risk scores among patients with different tumor stages and different sexes, we found that patient risk scores increased significantly with the stage of TNM, the key indicator of tumor progression (**Figure 2E**). However, patient risk scores were independent of sex.

**Figure 3:**
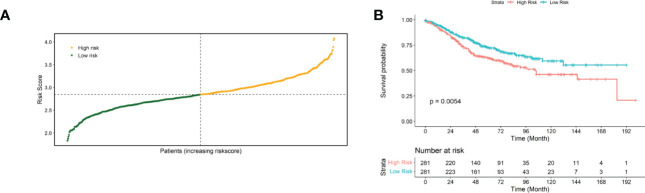
Validated risk scores for the GSE40967 dataset. **(A)** Risk score distribution of GSE40967 patients. **(B)** KM curves for high- and low-risk groups of GSE40967 patients.

Then, the performance of the risk scores for predicting the prognostic risk of patients was validated using an external independent validation set. We divided patients into high-risk and low-risk groups based on the median risk score as the cut-off point (**Figure 3A**). The Kaplan-Meier survival curve results showed that patients with lower risk scores exhibited higher survival (**Figure 3B**), and there was a significant difference between the high- and low-risk groups (p=0.0054).

### 3.3 GSEA analysis between high- and low-risk groups

To further investigate the differences in functions and pathways between the tumors of the high- and low-risk groups, we performed gene set enrichment analysis on the data. The pathways represented by different lines of red are molecular pathways or functions that were significantly activated in the tumor tissues of patients in the high-risk group, while the pathways represented by different lines of blue are molecular pathways or functions that were significantly activated in the tumor tissues of patients in the low-risk group.

**Figure 4:**
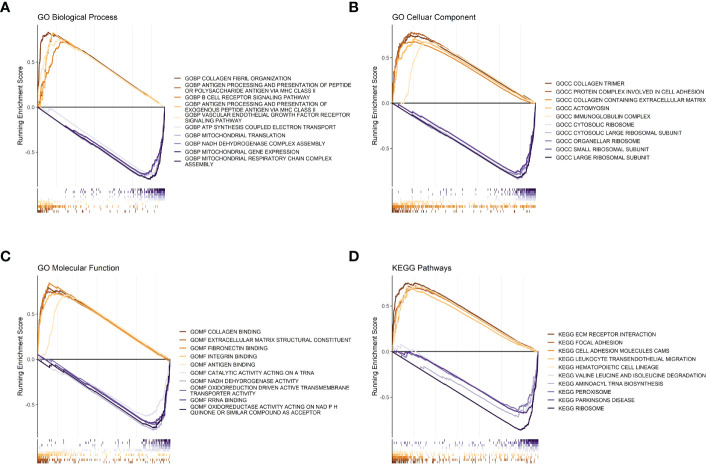
GSEA analysis between high- and low-risk groups. **(A)** Risk score GSEA: GO biological processes. **(B)** Risk score GSEA: GO cellular components. **(C)** Risk score GSEA: GO molecular functions. **(D)** Risk score GSEA: KEGG pathway.

Regarding biological processes, samples from the high-risk group were enriched in “Collagen fibril organization,” “antigen processing and presentation of peptide or polysaccharide antigen *via* MHC class II,” and “B cell receptor signaling pathway.” The samples from the low-risk group were enriched in “mitochondrial gene expression” and “mitochondrial respiratory chain complex assembly” (**Figure 4A**). Cellular Component analysis showed that “collagen trimer”, “protein complex involved in cell adhesion”, and “collagen containing extracellular matrix” were enriched in high-risk samples, whereas “large ribosomal subunit”, “small ribosomal subunit”, and “organellar ribosome” were enriched in low-risk samples (**Figure 4B**).

Regarding molecular functions, the high-risk group mainly showed enrichment in “collagen binding” and “extracellular matrix structural constituent”, while “oxidoreductase activity acting on NADPH”, “RNA binding”, and “oxidoreduction driven active transmembrane transporter activity” were enriched in the low-risk group (**Figure 4C**). Meanwhile, the KEGG gene sets showed enrichment in “ECM receptor interaction”, “focal adhesion”, and “cell adhesion molecules cams” in the high-risk group, while “ribosome”, “aminoacyl tRNA biosynthesis”, and “peroxisome” were enriched in the low-risk group (**Figure 4D**).

### 3.4 Risk score associated with the immune microenvironment after radiotherapy for CRC

**Figure 5:**
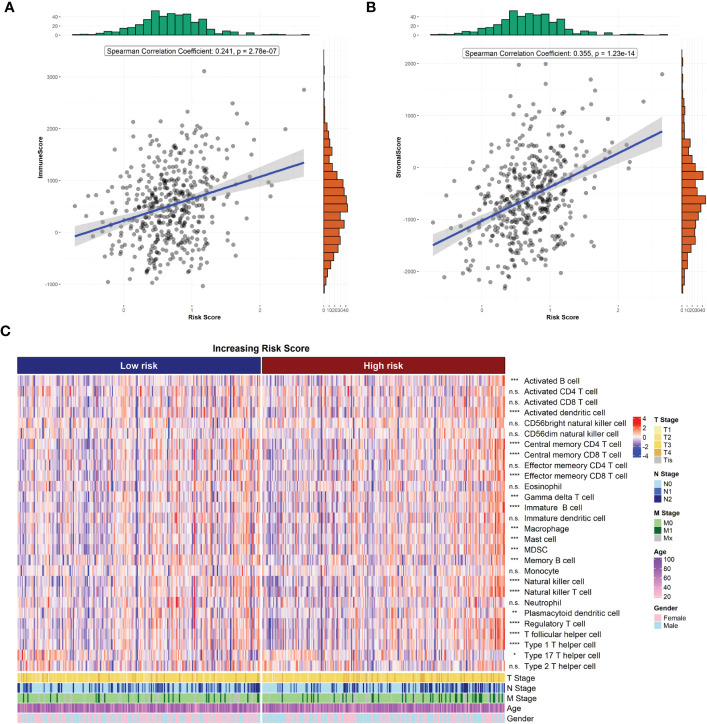
Risk score and tumor microenvironment. **(A)** Correlation of risk score with ESTIMATE immune score. **(B)** Correlation of risk score with ESTIMATE stromal score. **(C)** Heat map of immune cell infiltration.

The R package “ESTIMATE” was used to analyze the correlation between immune/stroma scores and risk scores. The results showed that both the immune score (**Figure 5A**) and stroma score (**Figure 5B**) were significantly higher in the high-risk group than in the low-risk group (both p<0.05). In addition, the ssGSEA method was used to estimate the infiltration of 28 immune cell species in samples from the high- and low-risk groups. The infiltration of 18 immune cell species significantly differed between the high- and low-risk groups (**Figure 5C**).

### 3.5 Predicting treatment sensitivity in patients with different risk scores

**Figure 6:**
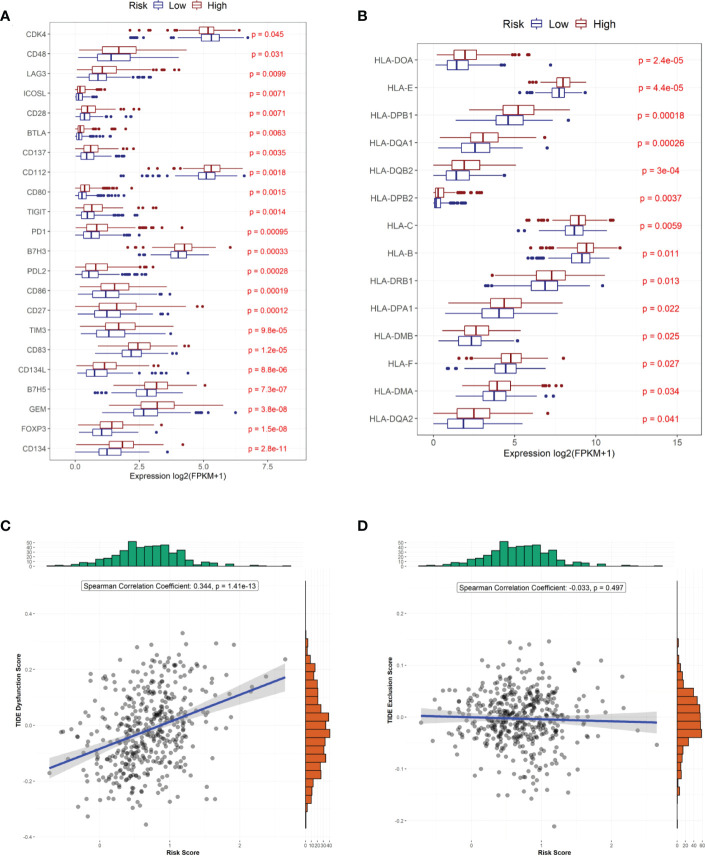
Predicted treatment sensitivity in patients with different risk scores. **(A)** Differential expression of immune checkpoint molecules. **(B)** Differential expression of HLA molecules. **(C)** Risk score versus immune disorder score. **(D)** Correlation between risk and immune rejection scores.

We also compared the expression of 34 immune checkpoint genes and 23 HLA family genes between the high- and low-risk groups and found that the expression of 22 immune checkpoints and 14 HLA family genes significantly differed between the two groups (**Figures 6A, B**). Among them, the top-three immune checkpoint genes and HLA family genes with the most significant differences were *CD134, FOXP3, GEM, B7H5, CD134L* and *HLA-DQAZ, HLA-DMA, HLA-F, HLA-DMB, HLA-DPA1*, respectively. To determine the potency of the risk score as a biomarker for predicting drug response in radiotherapy-tolerant patients with CRC, we assessed the drug-sensitivity of patients in the high- and low-risk groups to different antitumor drugs. The results show that a total of 44 drugs showed significant differences in sensitivity between the two groups. Among them, The ten most sensitive drugs in the high-risk group were Bortezomib, Epothilone B, Elesclomol, BEZ235, CGP-60474, Gemcitabine, QL-VIII-58, AZD7762, rTRAIL, and MG-132 (**Supplementary Table S4**). In terms of response to immunotherapy, patients in the low-risk group had lower TIDE dysfunction scores (**Figure 6B**); however, TIDE exclusion scores did not significantly differ from those in the high-risk group (**Figure 6D**).

### 3.6 Analysis of tumor mutation status in the high- and low-risk groups

**Figure 7:**
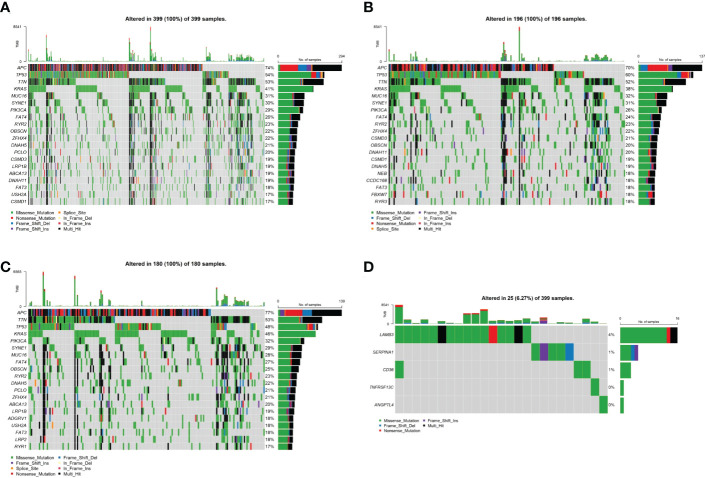
Analysis of tumor mutation status in high- and low-risk groups. **(A)** Top-20 mutation gene waterfall. **(B)** Top-20 mutation gene waterfall in the high-risk group. **(C)** Top-20 mutation gene waterfall in the low-risk group. **(D)** Model gene waterfall.

**Figure 8:**
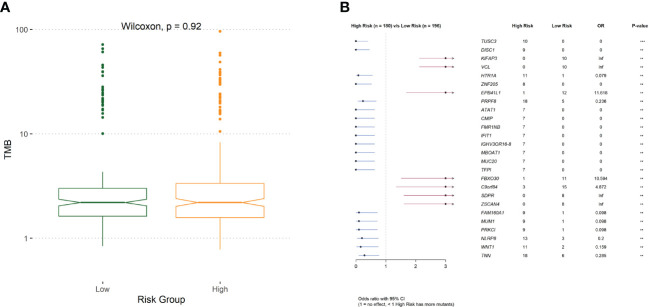
**(A)** The difference of tumor mutation burden between low- and high-risk group. **(B)**Comparison of risk for differentially mutated genes.

To investigate the mechanisms associated with poor prognosis in radiotherapy-tolerant CRC, we analyzed somatic mutations of all genes in the TCGA database. As shown in the waterfall figure (**Figure 7A**), *APC*, *TP53*, and *TTN* were the three most mutated genes in the COAD cohort. *KRAS* and *MUC16* were also identified as mutated genes in high-risk samples (**Figure 7B**). *FAT4* and *OBSN* were representative mutated genes in low-risk samples (**Figure 7C**). When comparing the mutation frequencies between the low- and high-risk group samples, more somatic mutations were observed in the high-risk group, but there was no significant difference (*p*=0.92) between the two groups (**Figure 8A**). The mutation frequencies of the 26 genes differed between the high- and low-risk groups (**Figure 8B**). Additionally, we visualized the mutation distribution of the five model genes; among them, *LAMB3* was the most mutated gene in the sample, and missense mutations were the most common mutation classification (**Figure 7D**).

### 3.7 Clinical prognostic model construction

**Figure 9:**
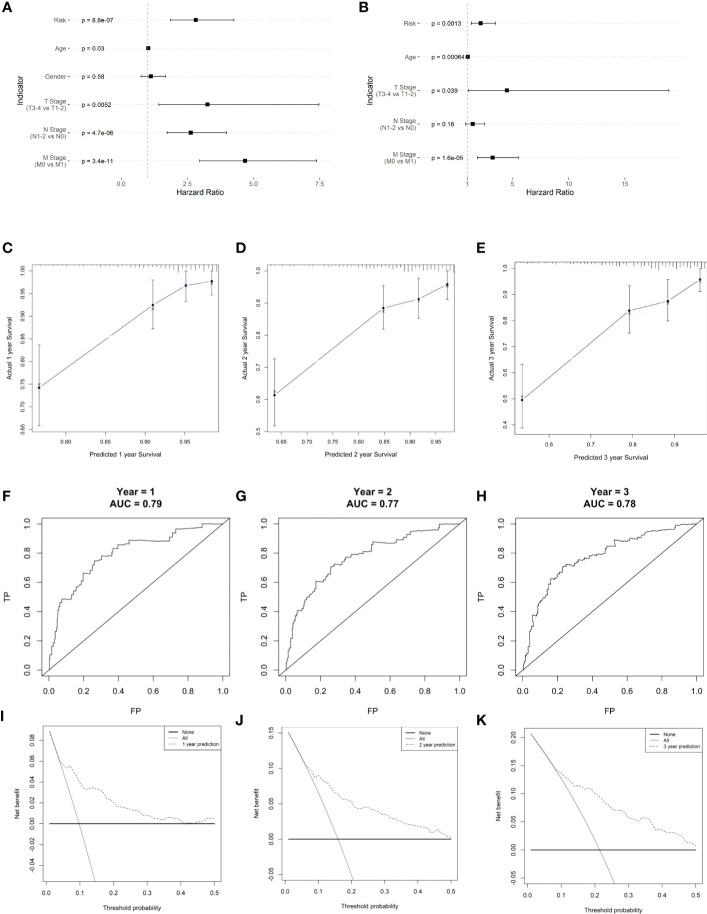
Clinical prognostic model construction. **(A)** Prognosis-related clinical indicators. **(B)** Forest plot of multifactorial Cox model. **(C–E)** 1-, 2-, and 3-year survival calibration curves, respectively. **(F–H)** Calibration curves for 1-, 2-, and 3-year survival rates, respectively. **(I–K)** ROC curves for 1-, 2-, and 3-year survival rates, respectively.

Risk score, age, and TNM stage were identified as significant predictors of prognosis, using univariate and multivariate Cox regression analyses (**Figure 9**). To predict the prognosis of each patient, a forest plot was created by integrating the risk score, age, and TNM stage, and we found that the hazard ratio of the risk score was higher than that of the T and N stages (**Figures 9A, B**). The calibration curve showed a high agreement between the risk score prediction and actual survival probability (**Figures 9C–E**). Next, we plotted ROC curves to examine the specificity and sensitivity of this prognostic model (**Figures 9F–H**). The time-dependent ROC curve showed that the AUC of the resulting prognostic model (based on the five-gene risk score and other clinical parameters, including age, sex, and tumor stages) was 0.79, 0.77, and 0.78 at 1, 2, and 3 years, respectively. The calibration curve showed high agreement between the risk score prediction and actual survival probability. In addition, the net benefit of this prognostic model was high, as shown by the DCA curve (**Figures 9I–K**).

## 4 Discussion

Radiotherapy is one of the most important treatments for patients with CRC (12). Radioresistance is the crucial cause of poor therapeutic outcomes in colorectal cancer. However, the underlying mechanism of radioresistance in colorectal cancer is still poorly defined. Therefore, there is an urgent need to identify genes associated with radioresistance that can accurately predict the therapeutic effect and prognosis of patients with CRC. Herein, we established a radioresistant colorectal cancer cell line and performed transcriptomics analyses to search for the underlying genes that contribute to radioresistance and investigate its association with the prognosis of CRC patients.

We found significant differences in mRNA between the HCT116^RR^ and HCT116^WT^ cell lines. GO enrichment analysis showed that the candidate genes were mainly enriched in extracellular matrix structural constituents and extracellular matrix binding, and KEGG enrichment analysis showed that the candidate genes were mainly enriched in focal adhesion and ECM-receptor interactions. The ECM-receptor interaction pathways were the most upregulated gene-enriched signaling pathways. ECM is a non-cellular component of tissues that supports cell adhesion, which is composed of different insoluble structural components such as collagen, elastin, proteoglycan, and glycoprotein (13). ECM can interact with a variety of receptors on tumor cells to regulate biological processes such as tumor shedding, migration, adhesion, and intercellular communication (14, 15). Radiotherapy can trigger cells within the tumor microenvironment to release enormous amounts of cytokines and chemokines to remodel the ECM, leading to tumor cells developing radioresistance (16). For colorectal cancer, studies have demonstrated the critical role of the ECM-receptor regulatory network in tumor development and metastasis (17). Therefore, high-quality prognostic indicators may be screened for in the ECM-receptor interaction pathway.

In further searches for key genes whose regulation affects CRC prognosis, we identified five signature genes, *TNFRSF13C, CD36, ANGPTL4, LAMB3, SERPINA1* using the one-factor Cox-LASSO method. Among them, *SERPINA1* is a protective factor for CRC, while *CD36, LAMB3, ANGPTL4* and *TNFRS13C* are all risk factors for CRC. These results are consistent with those of previous studies showing that the upregulation of these genes promotes proliferation and metastasis properties of CRC (18–20). Radiation-induced inflammation could trigger the overexpression of *CD36*. Previous studies have found that *CD36* promotes CRC metastasis by upregulating MMP28 and increasing E-calmodulin cleavage, which may be an important reason for the stronger invasion and metastasis ability of radioresistant cells (20). Similarly, it has also been shown that the overexpression of *LAMB3* in CRC is associated with tumor metastasis and poor prognosis, of which the mechanism is mainly through the AKT–FOXO3/4 axis to the pro-tumorigenic role (19). Moreover, *ANGPTL4* is associated with tumor metastasis and angiogenesis, and can promote CRC progression and metastasis by activating STAT1 and promoting trans activation of NOX4 (18, 21). Taken together, these studies demonstrate that these signature genes induced by irradiation may contribute to the development and progression of CRC after radioresistance by regulating cancer cell migration and invasion.

On this basis, we established a risk model using the signature genes to classify patients with CRC into high- and low-risk groups and performed survival analysis. Our results showed that the risk model was able to predict the prognosis of CRC patients. Subsequently, we performed GSEA on the high- and low-risk groups. GSEA is a method that uses genes from a pre-defined gene set to assess distribution trends in a gene list ranked with phenotypic relevance to judge their contribution to phenotype. We found that the high-risk group was mainly involved in the cell adhesion- and extracellular matrix-related pathway, while the low-risk group was mainly involved in the ribosome- and oxidoreduction-related pathway. These pathways are widely discussed in other reviews, especially the enrichment of “collagen containing extracellular matrix” in high-risk groups, which highlights that the remodeled ECM is an important contributing factor to malignant progression and resistance-to-therapy of tumors (22).

Fibrillar collagen is the main component of ECM. Cells embedded into fibrillar collagen interact with it through their surface receptors to exchange information with the outside world. Recent studies indicated that fibrillar collagen is upregulated in many cancers and that specific collagen fiber organization patterns are associated with disease stage, prognosis, treatment response, and other clinical features (23, 24). In colon tumor tissue, the expression of type 1 collagen is significantly higher than in normal tissue, and patients with a high density of type 1 collagen generally have a poor prognosis (25). Studies showed that type 1 collagen binds to integrins, such as α1β1, α2β1, α10β1, and α11β1, to enhance the stemness of colon carcinoma cells and promote CRC progression and metastasis (26). Furthermore, the receptor tyrosine kinases discoidin domain receptors DDR1 and DDR2 are also involved in type 1 collagen-mediated invasion and metastasis of colon carcinoma (27, 28). In addition, collagen levels and organization changes can also lead to several pathological consequences. Studies showed that aligned collagen increases stromal density and intra-tumor fluid pressure, which may impede the transport of therapeutic agents to tumor targets (29). This suggests that genes associated with “collagen fibril organization”, which were enriched in the high-risk group, may interfere with tumor cell metastasis after radiotherapy by affecting the density of collagen in the tumor. However, the question of how these ECM-associated proteins contribute to radiotherapy tolerance in colorectal cancer still needs to be further explored.

The ESTIMATE algorithm scores immune and stromal cells of tumor tissues. A higher ESTIMATE score indicates a higher tumor heterogeneity in the corresponding fraction. We found significant differences in ESTIMATE immune scores between the high- and low-risk groups, indicating that there was a higher tumor heterogeneity and higher degrees of malignancy in the high-risk group. In addition, we also estimated the difference of 28 immune cells infiltrated in samples from the high- and low-risk groups. Our results showed that there was a significant difference in the infiltration of 18 immune cell species between the two groups, including activated B cells, activated dendritic cells, gamma delta T cells, macrophages, myeloid-derived suppressor cells, and natural killer T cells. Studies show that the composition and number of immune cells in tumor tissue have a significant impact on tumor progression. An abnormal number of immune cells is significantly associated with poor prognosis in patients with CRC (30). Moreover, the paucity of immune cells also contributes to tolerance against immunotherapy and radiotherapy (31, 32). Thus, we hypothesized that radioresistance genes may also affect the prognosis of CRC.

We further analyzed the different responses to immunotherapy between the high- and low-risk groups. We observed significant differences in the expression of 22 immune checkpoints, and 14 HLA family genes significantly differed between the high- and low-risk groups, suggesting that the use of different immune checkpoint inhibitors may be appropriate for patients in different risk groups. The TIDE score is used to evaluate immune dysfunction and rejection within tumor tissue and can be used as a predictor of immunotherapy response. A higher TIDE prediction score represents a higher probability of immune escape. Therefore, studies on the correlation between risk score and TIDE can analyze the association between risk scores and the tumor immunotherapy effect. In our study, we found that patients in the high-risk group had higher TIDE dysfunction scores, indicating that patients in the high-risk group were less likely to benefit from immunotherapy, while patients in the low-risk group were more likely to respond to immunotherapy. Subsequently, we analyzed tumor mutations in the COAD cohort. The tumor mutation burden (TMB) has been identified as a biomarker of immunotherapy response, and a higher TMB predicts higher benefits of immunotherapy. In our study, we found that a total of 26 genes showed differences in mutation frequency between the high- and low-risk groups, which may be the main reason for the poor prognosis in the high-risk group. Furthermore, we investigated the potential mechanisms by which the characteristic genes regulate radioresistance in CRC. The clinical distribution of risk scores was analyzed, and we found that the patients’ risk scores were independent of sex but increased significantly with progressing TNM stages.

Taken together, we screened genes associated with radioresistance using sequencing data from HCT-116WT and HCT-116RR cells, and built a risk score model with five radioresistance genes, including *TNFRSF13C, CD36, ANGPTL4, LAMB3*, and *SERPINA1*. This risk score model showed favorable performance in prognosis prediction after radiotherapy for CRC. It also revealed the relevant mechanisms by which radioresistance genes regulate the prognosis of CRC. These results provide an important theoretical basis for subsequent biomarker research or drug target development.

## Data availability statement

The datasets presented in this study can be found in online repositories. The names of the repository/repositories and accession number(s) can be found in the article/**Supplementary Material**.

## Author contributions

HQ: Writing - original draft. HL and QX: Writing - review & editing. WS and HZ: Visualization. XZ: Conceptualization. SZha: Conceptualization. SZhu: Supervision. HW: Supervision, funding acquisition. All authors contributed to the article and approved the submitted version.
