# Supplementary material for: Prognostic risk analysis related to radioresistance genes in colorectal cancer
Source: Front Oncol. 2023 Jan 18;12:1100481. doi: 10.3389/fonc.2022.1100481 (PMC9890073; doi:10.3389/fonc.2022.1100481)
Supplement: Supplementary file 1 [file DataSheet_1.docx]

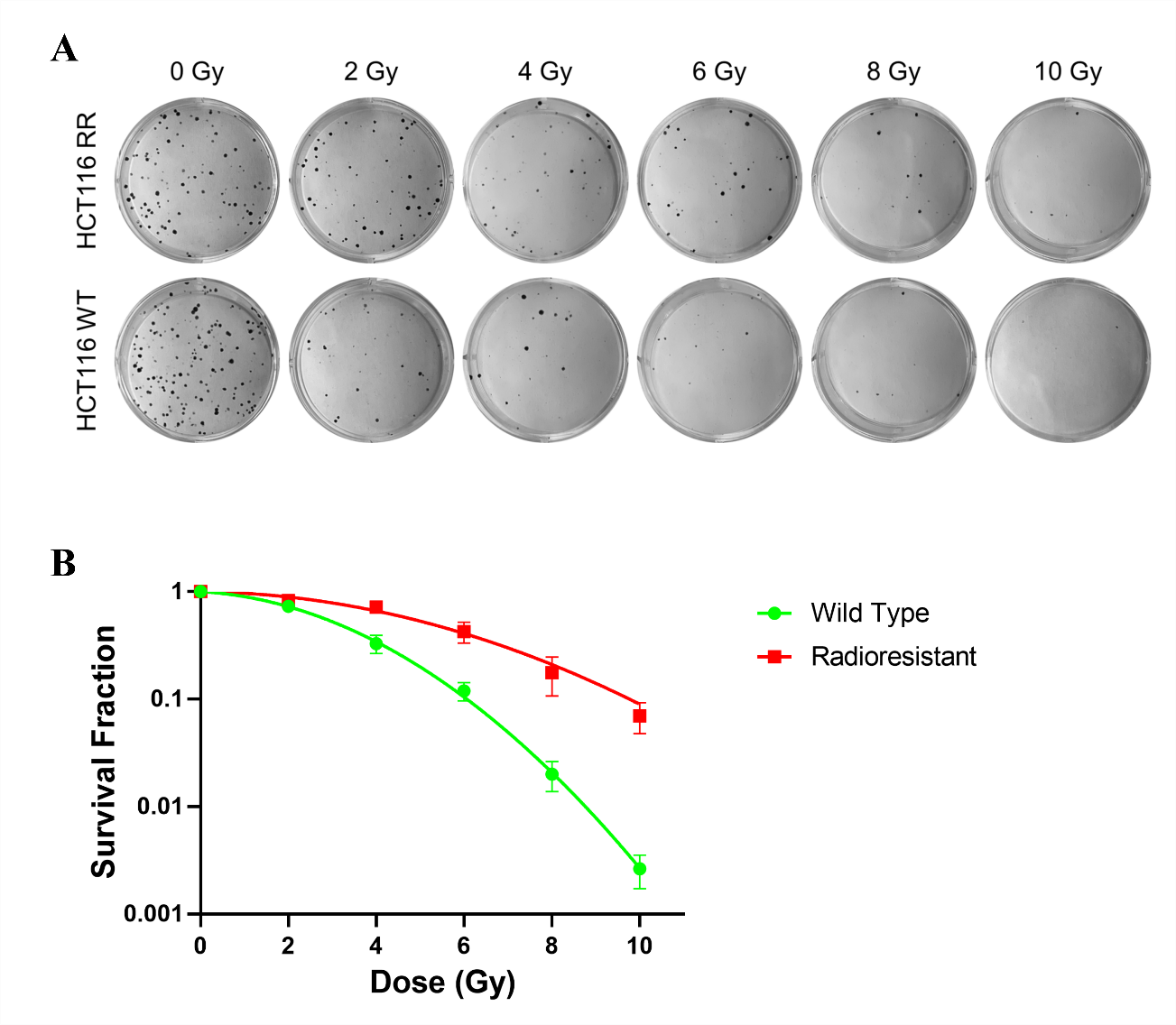


**Figure S1. The clone formation assay to verify the radioresistance between the wild-type and resistant HCT116 cell line.** (A) Wild-type and resistant HCT116 cell line were subjected to radiation clonogenic assays with the indicated radiation doses (2, 4, 6, 8 and 10 Gy). (B) The survival fraction between wild-type and resistant HCT116 cell line after irradiation.

| **Figure S2. The R package Version** | | |
| --- | --- | --- |
| **R package** | **Version** | **Application** |
| biomaRt | 2.50.3 | Get the location of the gene in the genome |
| ComplexHeatmap | 2.10.0 | Heatmap |
| clusterProfiler | 4.2.2 | GO and KEGG enrichment analysis |
| DESeq2 | 1.34.0 | Differential expression analysis |
| estimate | 1.0.13 | ESTIMATE score |
| GEOquery | 2.62.2 | Get GEO data |
| ggplot2 | 3.3.5 | Plot |
| ggstatsplot | 0.9.1 | The scatter plot |
| glmnet | 4.1-3 | LASSO modeling |
| GSVA | 1.42.0 | ssGSEA analysis |
| IOBR | 0.99.9 | TIDE score |
| org.Hs.eg.db | 3.14.0 | Gene ID conversion |
| OmicCircos | 1.32.0 | Circos plot |
| rms | 6.2-0 | Nomogram construction |
| survival | 3.3-1 | Survival analysis |
| survminer | 0.4.9 | KM survival curve |
| survivalROC | 1.0.3 | Prognostic efficiency and ROC curve |
| VennDiagram | 1.7.1 | Venn diagram plots |
