# Supplementary figures and images for: Prognostic risk analysis related to radioresistance genes in colorectal cancer
Source: Front Oncol. 2023 Jan 18;12:1100481. doi: 10.3389/fonc.2022.1100481 (PMC9890073; doi:10.3389/fonc.2022.1100481)

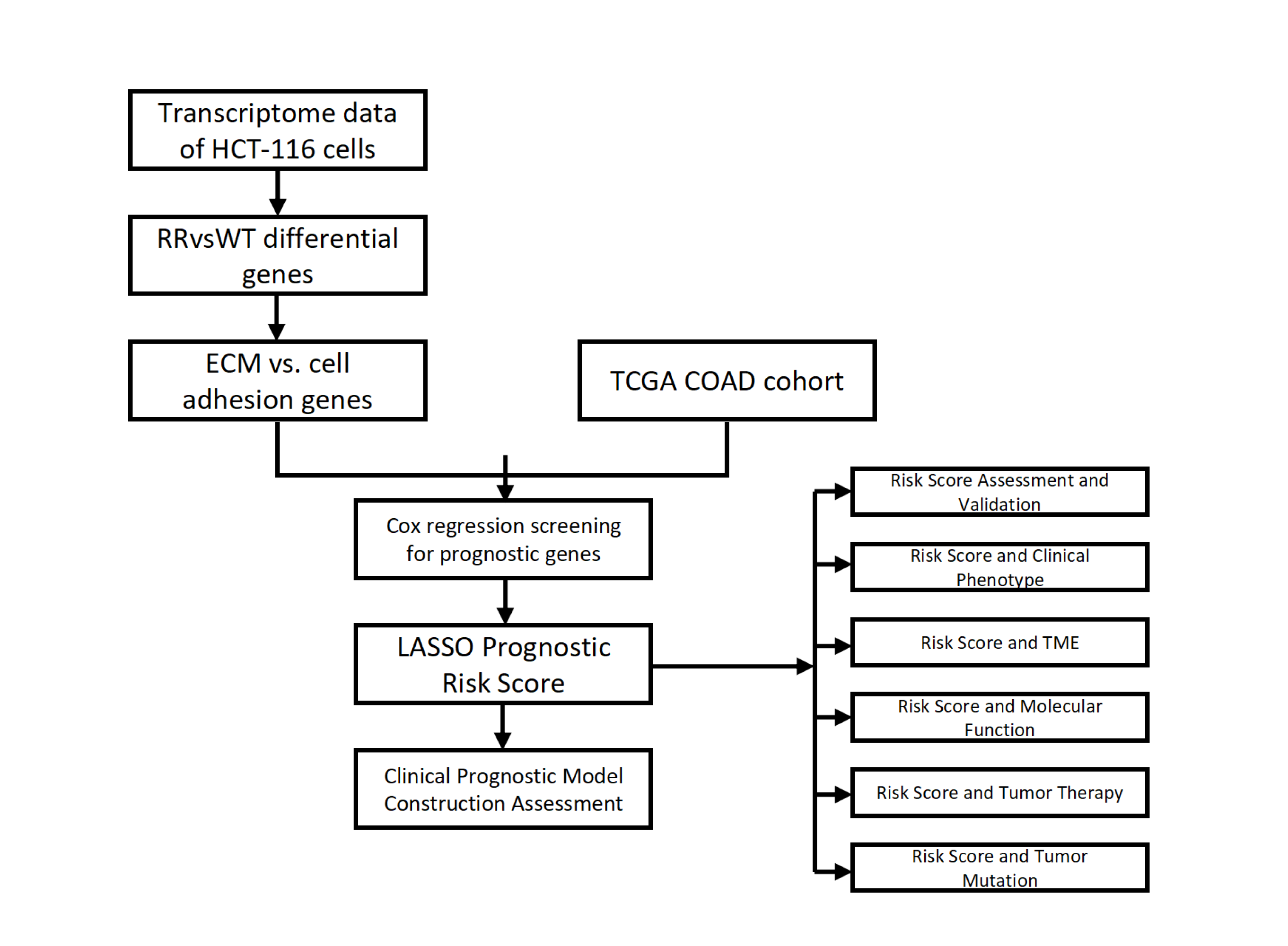

Supplement: Supplementary file 3 [file Image_1.tif]
